# Teacher stress and burnout in Australia: examining the role of intrapersonal and environmental factors

**DOI:** 10.1007/s11218-022-09686-7

**Published:** 2022-02-25

**Authors:** Annemaree Carroll, Kylee Forrest, Emma Sanders-O’Connor, Libby Flynn, Julie M. Bower, Samuel Fynes-Clinton, Ashley York, Maryam Ziaei

**Affiliations:** 1 Faculty of Humanities and Social Sciences, The University of Queensland, St Lucia, Brisbane, QLD 4072 Australia; 2 School of Education, The University of Queensland, Brisbane, Australia; 3 Centre for Advanced Imaging, The University of Queensland, Brisbane, Australia; 4 Rotman Research Institute, Baycrest, Toronto, Canada

**Keywords:** Teacher, Stress, Burnout, Workload, Emotion regulation, Subjective well-being

## Abstract

Concerns regarding high rates of teacher stress and burnout are present globally. Yet there is limited current data regarding the severity of stress, or the role of intrapersonal and environmental factors in relation to teacher stress and burnout within the Australian context. The present study, conducted over an 18-month period, prior to the COVID pandemic, surveyed 749 Australian teachers to explore their experience of work-related stress and burnout; differences in stress and burnout across different demographic groups within the profession; as well as the contributing role of intrapersonal and environmental factors, particularly, emotion regulation, subjective well-being, and workload. Results showed over half of the sample reported being very or extremely stressed and were considering leaving the profession, with early career teachers, primary teachers, and teachers working in rural and remote areas reporting the highest stress and burnout levels. Conditional process analyses highlighted the importance of emotion regulation, workload and subjective well-being in the development of teacher stress and some forms of burnout. Implications for educational practice are discussed.

## Introduction

Teaching is an extremely rewarding profession; however, it is also recognised as highly stressful and demanding. High rates of teacher occupational stress have been documented globally (e.g., Bottiani et al., 2019; Herman et al., 2018) and within Australia (OECD, 2020a), the context of the present study. Findings from the Teaching and Learning International Survey (TALISFreeman et al., 2014; OECD, 2014, 2020a) indicate that globally, high levels of administrative work are a notable source of stress for teachers, more so than long hours teaching in the classroom. According to TALIS (Freeman et al., 2014; OECD, 2014), teachers’ work in Australia is like other countries, with teachers on average spending 19 h teaching, 7 h planning, and 5 h marking per week. The number of hours working per week for Australian teachers, however, is 5 h higher than other countries, with an average of 43 h per week.

The COVID-19 pandemic has also heightened the stress of teachers world-wide with teachers becoming essential frontline workers in support of student learning and rapidly developing and delivering online materials to students remotely (Allen et al., 2020). School closures have been witnessed by 188 countries, affecting more than 1.7 billion children and adolescents, and their families (OECD, 2020b). With the closing and reopening of schools repeatedly occurring in many countries since March 2020, teachers have carried the burden of rapidly upskilling in digital literacy, while also having concerns for the engagement, motivation, and well-being of their students (Hoffman & Miller, 2020).

According to the Transactional Model of Stress and Coping, stress arises when there is a perceived discrepancy between the demands placed on an individual and their resources (e.g., time, ability) to successfully meet these demands (Sapolsky, 2004). Consistent exposure to occupational stress can lead to decreased job satisfaction, mental health problems, and may result in burnout and decisions to leave the profession (Brackett et al., 2010; Wang et al., 2015). According to findings by Goddard and Goddard (2006), there is a strong association between intention to leave the profession and teacher burnout, and subsequently high rates of attrition by early career teachers across numerous countries worldwide. As such, it is imperative to understand how and why teacher stress arises to make informed decisions about prevention and intervention approaches. The present study sought to determine the current prevalence of stress and burnout in a sample of Australian teachers, and to investigate which groups of teachers face the most stress. It also aimed to develop a deeper understanding of intrapersonal and environmental factors that may contribute to the development of teacher stress and burnout, and that may be targeted to help improve the work lives of teachers, with a particular focus on emotion regulation, subjective well-being and workload.

### Teacher stress and burnout

Teacher stress has been defined as “the experience by a teacher of unpleasant, negative emotions, such as anger, anxiety, tension, frustration or depression, resulting from some aspect of their work” (Kyriacou, 2001, p. 28). Burnout, on the other hand, has been conceptualised as “a state of physical, emotional, and mental exhaustion that results from long-term involvement in work situations that are emotionally demanding” (Schaufeli & Greenglass, 2001, p. 501). It has been consistently demonstrated that chronic stress is implicit in the development of burnout (e.g., Brackett et al., 2010; Vesely et al., 2013). Stress itself is believed to develop from an interplay of both environmental and intrapersonal factors (Sapolsky, 2004). Environmental factors that are frequently cited as sources of teacher stress include external demands such as excessive workload, time pressure, lack of resources, paperwork, student behaviour, organisational factors (e.g., level of leadership support, school climate), and scrutiny around teacher effectiveness (Carroll, Flynn, et al., 2021; De Nobile, 2016; Feltoe et al., 2016; Howard & Johnson, 2004; Skaalvik & Skaalvik, 2015; van Droogenbroeck & Spruyt, 2015). Relatively less focus has been placed on the importance of intrapersonal factors which may mitigate or accentuate stress, such as emotion regulation (e.g., Vesely et al., 2013), subjective well-being (Renshaw et al., 2015), cognitive appraisals (e.g., Chang, 2013), and personality factors (e.g., Kokkinos, 2007).

While much previous research has been conducted globally, mixed results have been proffered regarding the relative severity of stress, and which subsets of teachers are most at risk. For example, teachers were among six occupations found to report higher than average stress in a large survey of individuals from 26 occupations (Johnson et al., 2005), supporting the notion that teachers face more stress than the general public. Unfortunately, this study did not report details of sample characteristics, nor were the results of statistical significance. In a review of literature regarding the severity of teacher stress, van Droogenbroek and Spruyt (2015) highlighted methodological and statistical limitations within much of the research, such as the lack of a clear definition of stress with comparison across studies being difficult, the lack of specificity around educator type (e.g., primary teacher, secondary teacher, leadership team), the use of non-standardised measures, and the administration of one-item tests for stress, anxiety and depression without the severity of issues being identifiable.

Inconsistent results have been reported when looking at stress within the teaching population. For example, several studies indicate that early career teachers are at high risk of experiencing stress and burnout and that this is rising both within Australia and internationally (De Nobile & McCormick, 2010; Lau et al., 2005; Plunkett & Dyson, 2011). However other researchers argue that the risk for burnout does not differ according to experience (Chan et al., 2010; Chang, 2013). Across school levels, some research indicates that primary school teachers experience greater stress and burnout than high school teachers (Chan et al., 2010; Timms et al., 2006) while research from the OECD (2020a) indicates opposing trends across different countries suggesting no significant overall differences across educational levels. Rajendran et al. (2020) found no differences in levels of emotional exhaustion and burnout across primary and secondary teachers, while De Nobile and McCormick (2010) did not delineate between primary and secondary teachers but found classroom teachers to have greater stress than any other educator type. Finally, some preliminary evidence suggests that teachers in urban areas reported more stress compared to rural teachers, however no differences were found in the degree of burnout between these groups (Abel & Sewell, 1999). With such inconsistencies in research findings, no firm conclusions can yet be drawn about which Australian teachers may be most at-risk of experiencing stress and burnout, emphasizing the need for broad scale, current data.

### The effect of workload on stress and burnout

Workload is one of the most frequently cited sources of teacher stress, particularly as the profession experiences a process of intensification (i.e., an expansion of the teaching role to meet greater accountability demands; see Ballet & Kelchermans, 2009 for review). The concept of workload and what it encompasses for teachers is complex and multifaceted; reflecting the multiple roles teachers inhabit in their daily work lives. For example, Van Droogenbroeck et al. (2014) argue that workload needs to be distinguished between teaching related and non-teaching related tasks. Findings from their study confirmed that teaching-related workload combined with interpersonal relationships between the teacher and their students, had one of the strongest effects on the experience of emotional exhaustion. In a recent study on a group of Finnish teachers (n = 149), Salmela-Aro et al. (2019) identified two overarching teacher profiles: highly engaged (30%) and engaged-burnout (70%). Higher workload and larger class sizes were the main contributing factors for teachers in the “engaged-burnout” group. Although some key differences between work-related demands (i.e., workload) and personal resources (i.e., resilience) emerged between these two profiles, the authors concluded that the primary factors underpinning the potential experience of burnout for these teachers were largely school-level organisational problems (e.g., increases in class size, new national curriculum reforms, fast digitalisation, and Government funding cuts). In contrast, findings from Ryan et al. (2017) suggested that teachers were more likely to stay in their role if stressors constituted more externalised demands (e.g., workload) compared to internal factors (e.g., coping styles). Rajendran et al. (2020) found that both workload and student misbehaviour as well as work-family conflicts indirectly related to intent to leave the teaching profession and was mediated through emotional exhaustion. Considering this divergence of findings, the present study provides further insight into the role of workload in Australian teachers’ experience of stress and burnout.

### The role of emotion regulation in stress and burnout

Emotion regulation is a key intrapersonal capability which acts to accentuate or protect against the cumulative effects of stress (Chang, 2013; Jennings & Greenberg, 2009; Vesely et al., 2013). Emotion regulation is a learned skill, conceptualised as an individual’s ability to detect and identify emotional states in themselves or others, then employ strategies that up-regulate, sustain, or down-regulate these emotions to achieve a desired outcome (Brackett et al., 2010). There is strong empirical evidence demonstrating that deficits in emotion regulation are associated with the development and maintenance of mental health problems (including anxiety, depression, and many other disorders); and that enhancing effective emotion regulation skills is a promising way of fostering or restoring mental health (Berking & Whitley, 2014). In a teaching context, which involves constant management of interpersonal relationships, teachers who possess greater emotion regulation capabilities are predicted to experience less stress and burnout (Gross, 2002; Montgomery & Rupp, 2005). Interpersonally, teachers with sound emotion regulation ability are likely to recognise emotions in their students, predict the associated cognitions and behaviour, and choose an appropriate response that meets the needs of their students (Jennings & Greenberg, 2009; Sutton et al., 2009). At an intrapersonal level, the ability to recognise emotions such as anger or frustration within themselves, and choose whether to express this, is also beneficial in maintaining good student–teacher relationships and promoting teachers’ sense of self-efficacy (i.e., situational-specific self-confidence), buffering them against stress (Sutton et al., 2009). While good emotion regulation is likely to buffer against stress, ineffective emotion regulation strategies, such as suppressing or avoiding/faking emotions, have been associated with emotional exhaustion among teachers (Chang, 2013; Tsouloupas et al., 2010).

### Subjective well-being as a buffer against stress

Subjective well-being has been identified as one of the critical dimensions of psychological well-being (Su et al., 2014) and an important aspect of teachers’ successful and healthy functioning at work (Renshaw et al., 2015). Positive subjective well-being has been described as a heterogeneous category involving personal evaluations, such as life satisfaction or the perception of one’s moods and emotions as generally positive and manageable (Diener & Chan, 2011). Positive affect and subjective well-being appear to buffer against stress, with individuals in one study who scored higher on positive affect and overall subjective well-being found to have smaller physiological responses to stress, including lower blood pressure and cortisol levels (Steptoe et al., 2005). At a behavioural level, those with higher subjective well-being also endorse using coping strategies that protect against stress such as exercising, healthy eating, and accessing social supports (Steptoe et al., 2009). In relation to teachers particularly, positive subjective well-being indicators have been empirically related to a range of valued educational outcomes, such as teaching effectiveness, better classroom climate and student well-being (see Renshaw et al., 2015). Factors that have been shown to predict teachers’ subjective well-being include the quality of social supports, leadership support, school climate (Granziera et al., 2021), self-efficacy, job satisfaction, innovative teaching practices (Katsantonis, 2020), and workload stress (Collie et al., 2012; OECDa, 2020). These relationships underscore the protective role that subjective well-being plays, enabling individuals to respond to workplace challenges positively. While it appears reasonable to infer that high subjective well-being would protect teachers against the effects of occupational stress, this assumption requires direct exploration especially when considering the emotion regulation of teachers.

### The present study

To date, little if any research has been conducted to examine the effect of workload, emotion regulation and subjective well-being in relation to Australian teachers’ stress and burnout. As such, the following research questions were addressed: (1) How is stress experienced for Australian teachers across different job-related teacher characteristics, including career stage, educator type, and geographic location? (2) What is the role and relative importance of environmental and intrapersonal factors, specifically emotion regulation, workload, and subjective well-being, in the experience of teacher stress and burnout in Australia?

To address the first research question, a full factorial multivariate analysis of variance (MANOVA) was conducted with perceived stress, work-related burnout, and student-related burnout as the dependent variables and career stage, educator type, and geographic location, as fixed factors. To address the second research question, the present study posited subjective well-being to be a primary predictor of interest because of its role as a protective factor against stress and burnout directly (Granziera et al., 2021) and against negative workplace experience such as perceived workload (Collie et al., 2012). Workload was also included as a mediator of both stress and burnout generally because of its close link to stress and burnout (Ballet & Kelchermans, 2009). Emotion regulation was included as a serial mediator because of its intermediary role between workplace challenges (workload) and stress (Chang, 2013; Jennings & Greenberg, 2009; Vesely et al., 2013). Finally, the relationship between stress and burnout led us to include perceived stress as a serial mediator in the analyses predicting both work-related burnout and student-related burnout.

## Method

### Participants

Seven hundred and forty-nine registered teachers and teaching professionals throughout Australia participated in the present study. Teachers were recruited nationally via an advertisement for the study emailed to educators who subscribed to the Australian Research Council—Special Research Initiative Science of Learning Research Centre (SLRC) mailing list and via social media networks (e.g., Facebook, Twitter) at various time points across an 18-month period. The sample consisted of 625 females (83%) and 124 males (17%), most of whom were experienced teachers (66% having more than 10 years teaching experience) and middle aged (*M* = 44.27, *SD* = 11.29, Range = 22–75 years). The majority of the sample were located in an urban setting (69%). It is estimated that 1.9% of Australian teachers are located in remote or rural locations (Australian Bureau of Statistics, 2018; Willis & Grainger, 2020), suggesting that the present study has fairly represented this geographic class with 9% of respondents reporting teaching in a rural or remote region. These statistics concur with the data reported in the National Teaching Workforce Dataset (NTWD) report (Willett et al., 2014), suggesting that the current sample is demographically similar to the profession generally. The NTWD reports on data from 440,313 people that comprise the Australian teaching workforce. The report lists the median age of teachers in Australia as 44 years old, three quarters of which are female, and working in a major city (65%). Table 1 presents the demographic and professional characteristics of the sample.

**Table 1:** Demographic and professional characteristics of the sample

Characteristic	*n*	%
Gender		
Female	625	83
Male	124	17
Type of teacher		
Administration	95	13
Primary	270	36
Secondary	356	48
Other	25	3
Experience		
Early career	169	23
Mid-career	342	46
Late-career	238	32
Geographic region		
Urban	511	69
Regional	166	22
Rural/remote	67	9

### Measures

#### Stress

The *Perceived Stress Scale* (*PSS;* Cohen et al., 1983) is a widely used 10-item questionnaire measuring the degree to which situations in the respondent’s life are appraised as stressful. Respondents rate the frequency of thoughts and feelings during the past month on a 5-point scale ranging from 0 (never) to 4 (very often). Scores for all items are summed to form a total score, with higher numbers representing greater levels of Perceived Stress (Range = 0–40). The PSS has been reported to have sound psychometric properties in previous research (e.g., Santiago et al., 2020) and had good internal consistency in the present study, Cronbach’s *α* = 0.87.

Three further questions were used to measure general job-related stress and intention to leave the profession, following the approach used by Kyriacou and Sutcliffe (1978). The question, “In general, how stressful do you find being a teacher?” was rated on a 5-point Likert scale (1 = Not at all stressful to 5 = Extremely stressful). The question, “In the last month, have you considered leaving your current role due to stress or dissatisfaction?”, was rated “Yes” or “No”. Participants who responded affirmatively were asked, “How seriously have you considered leaving your current role?”, scored on a 5-point Likert scale ranging from 1 = Not at all to 5 = Extremely seriously.

#### Burnout

The *Copenhagen Burnout Inventory (CBI;* Kristensen et al., 2005) assesses symptoms of burnout for individuals working in the human service sector across three different domains: personal/general burnout (6 items measuring the degree of physical and psychological fatigue and exhaustion experienced by the individual, e.g., “How often do you feel tired?”), work-related burnout (7 items measuring the degree of physical and psychological fatigue and exhaustion that is perceived by the person as related to the individual’s work, e.g., “Do you feel burnout because of your work?”) and client/student-related burnout (6 items measuring the degree of physical and psychological fatigue and exhaustion that is perceived by the individual as related to the individual’s clients or students, e.g., “Do you find it hard to work with students?”). Relevant for the present study were the work-related burnout and client/student-related burnout scales. Responses are indicated on a five-point scale ranging from Never to Always, with scores for each subscale reported as averages. The CBI has been reported to possess satisfactory reliability and construct validity (e.g., Milfont et al., 2008). Internal consistency in the current study was high for both work-related burnout (α = 0.87) and client/student-related burnout (α = 0.88).

#### Workload

The *Teachers’ Perceived Context Scale* (*TPCS;* Bower & Carroll, 2014) is an 11-item scale developed to measure teachers’ perceptions of their workplace in the domains of student behaviour, workload, staffroom environment, and burnout. Respondents rate their degree of agreement with each item using a five-point scale with responses ranging from Strongly Disagree to Strongly Agree. The 3-item Workload subscale was used in the current study and was found to have satisfactory internal consistency (α = 0.65). Example items from the Workload subscale include, “I think my workload is reasonable”, and “I have sufficient time to prepare for my lessons as I would like to.”

#### Emotion regulation

*The Difficulties in Emotion Regulation Scale* (*DERS;* Gratz & Roemer, 2004) is a 36-item measure of emotion regulation capacities. The total DERS score was used for this study. Sample items include: “When I am upset, I feel guilty for feeling that way”; “I am attentive to my feelings”; “When I’m upset, I believe that I will remain that way for a long time”; “I am clear about my feelings”. Responses are scored on a 5-point Likert scale representing the frequency of behaviours (1 = Almost Never, 5 = Almost Always), with higher scores indicating greater emotion regulation difficulties. The DERS has been reported to possess good construct validity, test–retest reliability and internal consistency (e.g., Fowler et al., 2014) and this was consistent within the present study, α = 0.94 for all items.

#### Well-being

*The Comprehensive Inventory of Thriving* (*CIT;* Su et al., 2014) is a widely used measure of psychological well-being. Of relevance for the present study was the 9-item Subjective Well-being subscale. Respondents rate their level of agreement to each of the positively worded statements on a five-point scale (1 = Strongly Disagree, 5 = Strongly Agree) with higher scores indicating greater well-being. Example items include: “I feel positive most of the time”; “I am satisfied with my life”. The CIT has been reported to possess sound psychometric properties including convergent and divergent validity with existing measures of well-being and ill-health (Su et al., 2014). In the present study, the subjective well-being scale was found to have strong internal consistency (α = 0.94).

### Procedure

Approval to conduct the study was received from the Human Research Ethics Committee at the administering institution. An online survey platform was used to administer the questionnaire to maximise accessibility of the study to teachers across Australia while minimising the time burden placed on them (Ebert et al., 2018). A link to the survey was included in the advertisement and participants provided their informed consent to take part, prior to completing the 20-min survey. All responses were anonymous and confidential. The timing of advertisements was staggered across different periods of the school year to capture the range of high and low workload periods, although it should be noted that the study was conducted over an 18-month period, prior to the COVID pandemic. Data were compiled by Qualtrics and analysed using both IBM Statistical Package for Social Sciences version 24, and JASP (2021).

## Results

**Fig. 1:**
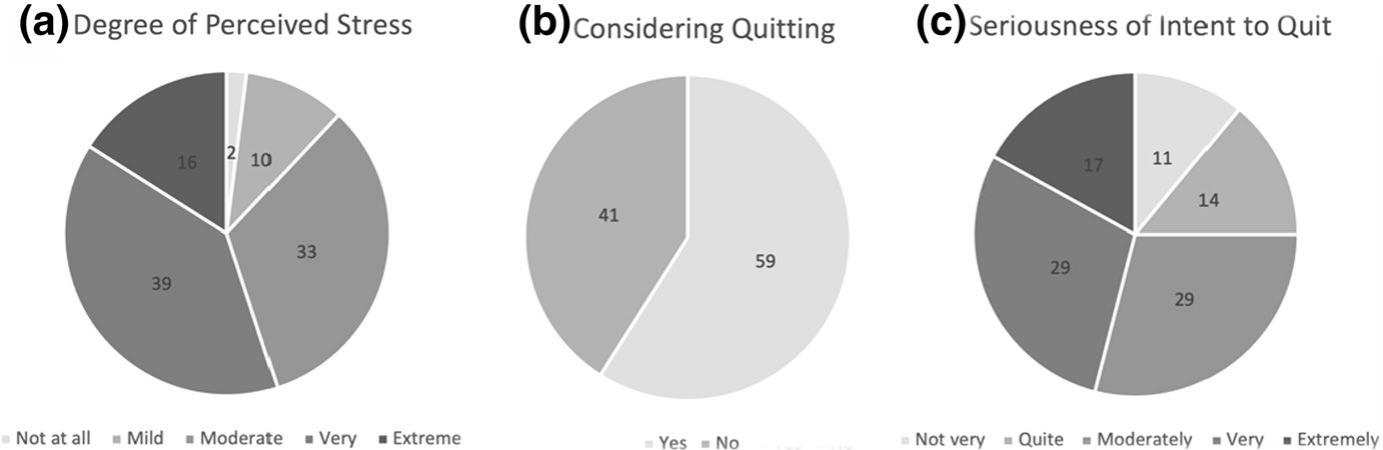
Percentage distributions of teachers’ responses to questions regarding: **a** Degree of Perceived Job-stress, **b** Considerations of Quitting Teaching, and **c** Seriousness of Intentions to Quits

A total of 941 surveys were returned. Fifty-five surveys were discarded due to incomplete information within the demographic section and missing survey responses. An additional 137 surveys were discarded due to incomplete responses to psychometric measures. In total, 749 surveys were included for analysis. As shown in Fig. 1, more than half of the respondents (55%; n = 413) rated their job as very or extremely stressful and 59% (n = 437) of respondents reported considering leaving their role in the past month due to stress or dissatisfaction. Of those who considered leaving, 75% (n = 328) reported that these thoughts were moderately to extremely serious. The non-completers differed from those who completed the survey in age (mean = 42.10 versus 44.27 years), gender and teaching experience (15.04 versus 17.66 years), whereby those who completed were significantly older (*t*(882) = − 2.06, *p* = 0.040), more experienced (*t*(837) = − 2.48, *p* = 0.014) and male (*t*(884) = − 3.52, *p* < 0.001).

### Stress and burnout profiles across teacher-related characteristics

In response to the first research question, the dispersion of perceived stress and both work-related and student-related burnout within groups of Australian teachers according to job-related characteristics was explored. Taking into consideration the significant correlations between perceived stress, work-related burnout, and student-related burnout, we conducted a full factorial MANOVA, with the following fixed factors: career stage, educator type, and geographic location, as well as their combined interaction terms. Please note that while observations were collected from additional educator types (i.e., pre-school, university and other), they were limited in number, hence the following analysis was confined to the following educator types: administration, primary and secondary. There were statistically significant differences in stress and burnout based on career stage *F*(6, 1368) = 3.25, *p* = 0.004; Wilk’s Λ = 0.972, ηp2 = 0.01), educator type *F*(6, 1368) = 6.98, *p* < 0.001; Wilk’s Λ = 0.941, ηp2 = 0.03), and geographic location *F*(6, 1368) = 3.98, *p* < 0.001; Wilk’s Λ = 0.966, ηp2 = 0.02), although there were no interaction effects between predictor variables (*p* > 0.335).

To systematically follow up the variability in perceived stress, and both work-related and student-related burnout, univariate ANOVAs with post-hoc comparisons were conducted. Results are presented in Table 2 including descriptive statistics. We found that perceived stress differed significantly for all factors of job-related characteristics. Post-hoc comparisons using the Tukey HSD test indicated that early career teachers reported significantly higher levels of perceived stress than mid- (*p* = 0.022) or late career teachers (*p* = 0.003). A similar pattern of elevated difficulty for early career teachers was found within the burnout scales as measured by the Copenhagen Burnout Inventory (CBI). Post hoc comparisons suggested that work-related burnout was significantly greater for early career teachers in comparison to late-career (*p* = 0.003) but not mid-career colleagues (*p* = 0.263), with no other differences found. With regard to student-related burnout, early career teachers reported significantly higher levels than did mid-career teachers (*p* = 0.007); no other differences were found.

**Table 2:** Descriptive statistics and univariate ANOVAs comparing stress and burnout across job-related characteristics

Test	Variable	Group	*N*	M (*SD*)	*F (df)*	*p*	*η*^2^
Perceived Stress	Career stage	Early career	169	21.62 (5.68)_*ab*_	3.372 (2, 686)	.035	0.02
		Mid-career	342	20.15 (6.12)_*a*_			
		Late career	238	19.62 (6.12)_*b*_			
	Educator type	Leadership	98	18.35 (5.93)_*cd*_	12.659 (2, 686)	< .001	0.04
		Primary	256	21.75 (5.65)_*ce*_			
		Secondary	356	19.98 (6.25)_*e*_			
		Preschool	14	19.36 (6.22)			
		University	20	18.95 (4.97)			
		Other	5	16.8 (3.9)			
	Geographic location	Urban	511	19.83 (5.93)_*f*_	5.829 (2, 686)	.00	0.02
		Regional	166	21.43 (6.27)_*f*_			
		Rural/remote	67	21.09 (6.14)			
Work-related Burnout	Career stage	Early	169	61.81 (18.29)_*b*_	3.832 (2, 686)	.022	0.01
		Mid	342	59.35 (18.97)			
		Late	238	55.64 (18.87)_*b*_			
	Educator type	Leadership	98	53.1 (17.64)_*cd*_	8.580 (2, 686)	< .001	0.02
		Primary	256	62.17 (18.14)_*c*_			
		Secondary	356	58.49 (19.19)_*d*_			
		Preschool	14	56.68 (23.06)			
		University	20	51.43 (17.97)			
		Other	5	45 (16.87)			
	Geographic location	Urban	511	57.56 (18.76)_*f*_	3.453 (2, 686)	.032	0.01
		Regional	166	61.62 (19.36)_*f*_			
		Rural/remote	67	60.07 (18.45)			
Student-related Burnout	Career stage	Early	169	40.46 (19.01)_*a*_	2.235 (2, 686)	.108	0.01
		Mid	342	34.86 (20.34)_*a*_			
		Late	238	36.43 (20.59)			
	Educator type	Leadership	98	27.59 (18.55)_*cd*_	12.892 (2, 686)	< .001	0.04
		Primary	256	39.44 (19.69)_*c*_			
		Secondary	356	37.57 (20.44)_*d*_			
		Preschool	14	32.86 (22.08)			
		University	20	33.54 (17.96)			
		Other	5	25 (18.87)			
	Geographic location	Urban	511	34.97 (18.91)_*fg*_	7.265 (2, 686)	< .001	0.02
		Regional	166	39.49 (22.13)_*f*_			
		Rural/remote	67	42.29 (23.71)_*g*_			

*a* indicates early- differed significantly from mid-career; *b* indicates that early- differed significantly from late-career; *c* indicates that Leadership differed significantly from Primary, *d* indicates that Leadership differed significantly from Secondary, *e* indicates that Primary differed significantly from Secondary; *f* indicates that Urban differed significantly from Regional, *g* indicates that Urban differed significantly from Rural/Remote

Significant between group differences in stress and burnout were also found among teachers working in different educator type roles. Post hoc comparisons using the Tukey HSD test indicated that primary teachers reported significantly more perceived stress compared to educators in leadership roles (*p* < 0.001) or secondary teachers (*p* = 0.001). Similarly, burnout levels were found to be significantly higher in both primary (work-related burnout: *p* < 0.001; student-related burnout: *p* < 0.001) and secondary teachers (student-related burnout: *p* = 0.030) compared to those reported by educators in leadership positions.

Finally, rates of stress and burnout were also found to differ significantly according to geographical location. Post-hoc comparisons indicated that teachers working in regional areas experienced greater levels of perceived stress (*p* = 0.012) and work-related burnout (*p* = 0.048) compared to teachers in urban areas. Likewise, the degree of student-related burnout was significantly greater for teachers in both regional (*p* = 0.039) and rural/remote areas (*p* = 0.029) in comparison to that reported by teachers in urban areas.

### Predicting stress and burnout

**Fig. 2:**
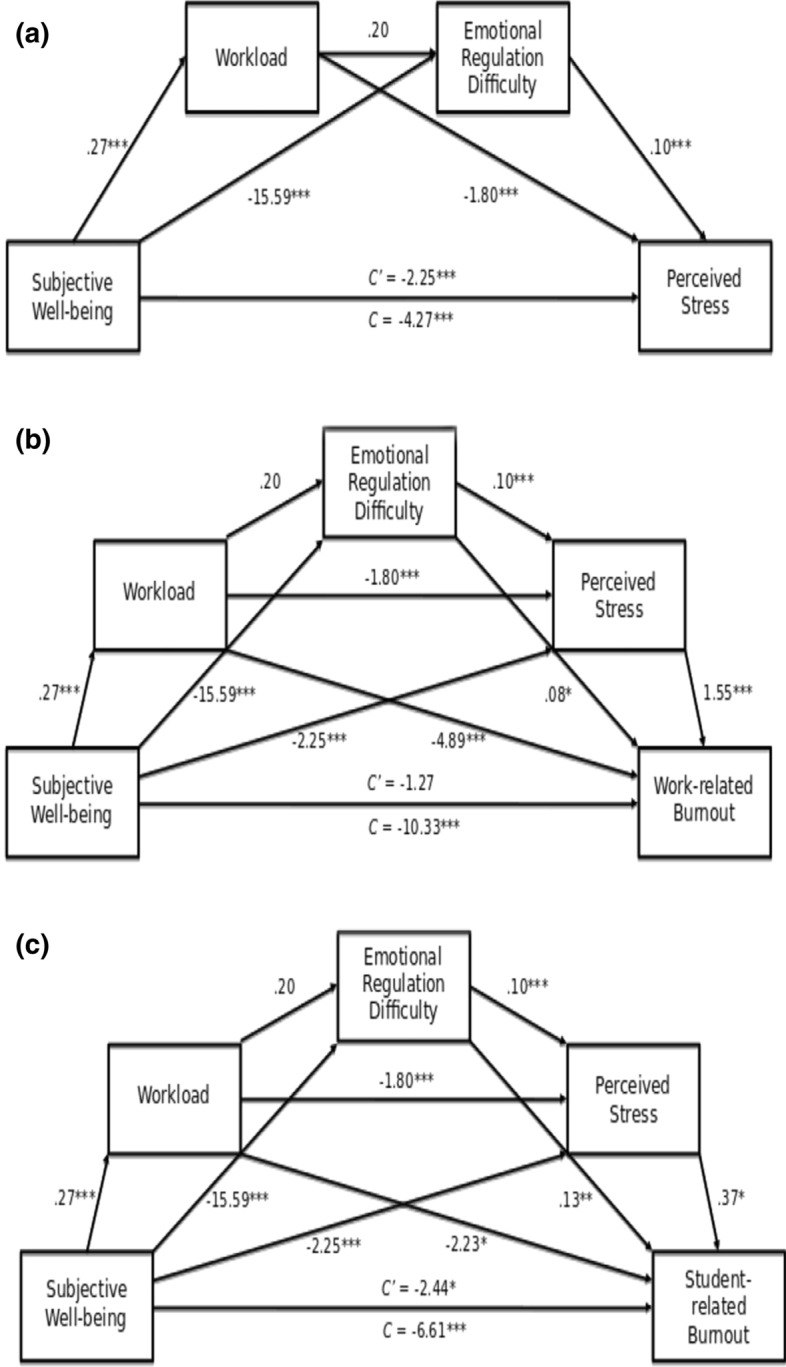
Three conditional process models determining relative importance of emotion regulation difficulties, workload, and subjective well-being on **a** Perceived Stress, **b** Work-Related Burnout And **c** Student-Related Burnout after Controlling for Gender, Years of Experience, Educator Type, and Geographical Location

In response to the second research question, three conditional process models were used to determine the relative importance of emotion regulation difficulties, workload, and subjective well-being, on perceived stress, work-related burnout and student-related burnout after controlling for gender, years of experience, educator type, and geographical location (see Fig. 2). Preliminary analyses were conducted to ensure no violation of the assumptions for this analysis; dummy variables were created for the categorical demographic characteristics, namely setting (using the urban setting as the dummy variable) and educator type (using administration as the dummy variable). The correlations between variables, presented in Table 3, show moderate to strong relationships between the predictor and outcome variables. All models were conducted using Hayes’ PROCESS macro (Hayes, 2018) in SPSS. Serial mediation analyses (model 6) were conducted, with 10,000 bias-corrected bootstrap samples, and our critical level of significance was set to a *p*-value of 0.05. Indices of indirect effects were considered statistically significant if the 95% confidence interval (CI), estimated using the bootstrap method, did not include zero.

**Table 3:** Correlations, means, standard deviations and skewness for all continuous variables

	Mean (SD)	Skewness (SE Skew)	1	2	3	4	5	6	7
1. Years Teaching +	17.66 (11.24)	0.42 (0.09)	–						
2. Subjective Well-being +	3.62 (0.81)	− 0.69 (0.09)	.02	–					
3. Workload	2.68 (0.86)	0.22 (0.09)	.03	.25***	–				
4. Difficulties in Emotion Regulation +	77.99 (21.83)	0.81 (0.09)	− .18***	− .54***	− .17***	–			
5. Perceived Stress	20.31 (6.06)	0.03 (0.09)	− .13***	− .56***	− .38***	.57***	–		
6. Work− related Burnout	58.73 (18.9)	− 0.2 (0.09)	− .15***	− .42***	− .45***	.44***	.68***	–	
7. Student-related Burnout +	36.62 (20.22)	0.36 (0.09)	− .13***	− .29***	− .23***	.28***	.31***	.51***	–

*p < .05, **p < .01, *** *p *< .001; + denotes skew, Spearman’s Rho reported accordingly

#### Predicting perceived stress

The overall regression model was significant, with 50% of variance in perceived stress accounted for by subjective well-being, workload and emotion regulation difficulties, *R*^2^ = 0.59, *F*(9, 656) = 73.65, *p* < 0.001. Figure 2A depicts the serial mediation model tested including the coefficients and standard errors for each path. The regression statistics are reported in Table 4.

**Table 4:** Standardised slopes and portion of variance in perceives stress and burnout explained in full models

Outcome	Final regression statistics	Variable	Standardised coefficient (*β)*	Part correlation (sr^2^)
Perceived Stress	*R*^2^ = .59, *F*(9, 656) = 73.65, *p* < .001	Gender	1.61**	0.11
		experience	− 0.03*	− 0.05
		Primary teacher	0.93	0.06
		Secondary teacher	− 0.31	− 0.01
		Regional teacher	0.68	0.04
		Rural/remote teacher	0.13	0.01
		Subjective well-being	− 2.25***	− 0.24
		Perception of workload	− 1.80***	− 0.23
		difficulties with emotion regulation	0.10***	0.29
Work-related	*R*^2^ = .52, *F*(10, 655) = 70.76, *p* < .001	Gender	0.02	0.03
		experience	− 0.07*	− 0.06
		primary teacher	0.01	0.01
		Secondary teacher	< 0.01	< 0.01
		Regional teacher	0.02	0.01
		Rural/remote teacher	− 0.01	− 0.02
		Subjective well-being	− 0.05	− 0.03
		perception of workload	− 0.22***	− 0.21
		Difficulties with emotion regulation	0.08*	0.07
		Perceived stress	0.50***	0.36
Student-related	*R*^2^ = .16, *F*(10, 655) = 13.19, *p* < .001	Gender	− 0.02	− 0.02
		experience	− 0.03	− 0.02
		Primary teacher	0.19***	0.13
		Secondary teacher	0.18**	0.12
		Regional teacher	0.06	0.06
		Rural/remote teacher	0.11**	0.09
		Subjective well-being	− 0.10*	− 0.05
		perception of workload	− 0.09*	− 0.10
		Difficulties with emotion Regulation	0.14**	0.10
		perceived stress	0.11*	0.09

Dummy variables are the following: Gender (male = 0, female = 1); Primary teacher (secondary/admin = 0, primary = 1); Secondary teacher (admin/primary = 0, secondary = 1); Regional teacher (urban/rural/remote = 0, regional = 1); Rural/remote teacher (urban/regional = 0, rural/remote = 1)

Overall, in the full model predicting perceived stress, emotion regulation difficulties (*b* = 0.10; CI [0.08, 0.19]) was found to be the strongest unique predictor of perceived stress, accounting for 9% of the overall variance, with subjective well-being (*b* = − 2.25; CI − 2.77, − 1.72) and workload (*b* = − 1.80; CI [− 2.20, − 1.39]) acting as the second and third strongest predictors, accounting for 6% and 5% respectively. Greater emotion regulation difficulties were associated with greater perceived stress, while having a manageable workload appeared to protect against stress. Gender (*b* = 1.60; CI [0.69, 2.51]) and years of experience teaching (*b* = − 0.03; CI − 0.07, − 0.003) were the only significant demographic characteristics, suggesting that being a female teacher, or being new to the profession was associated with higher levels of perceived stress.

The total *b* = − 4.27, *t*(666) = − 17.56, *p* < 0.001 and direct effects *b* = − 2.24, *t* (666) = − 8.44, *p* < 0.001 of subjective well-being on perceived stress were both significant, suggesting that partial mediation had occurred (Hayes, 2018). The total indirect effect of subjective well-being on perceived stress, representing the sum of the specific indirect effects, was significant, *b* = − 2.03; CI − 2.45, 1.63. Comparisons of the indirect effects suggested that, while the indirect pathway from subjective well-being to perceived stress through workload was significant, the best predictor of perceived stress was the indirect pathway of subjective well-being working through emotional regulation difficulties (see Table 5 for indirect pathway comparisons). Specifically, as subjective well-being increased, workload also increased, which led to a decrease in perceived stress. It had no significant influence on emotion regulation difficulties. Similarly, an increase in emotion regulation difficulties led to an increase in perceived stress. That is, despite predictions it appears that subjective well-being seems to impact perceived stress via both workload and emotion regulation difficulties, but these latter mediators are not serial.

**Table 5:** Indirect pathways, and pairwise comparisons of indirect Pathways with unstandardized coefficients (standard error) and bootstrapped confidence intervals

Outcome	Indirect pathways	Comparison	Coefficient (SE)	CI
Perceived Stress	SWB → Workload → Perceived Stress	Ind1^	− 0.48 (0.09)	− 0.67, − 0.31
	SWB → Emotion Recognition Difficulty → Perceived Stress	Ind2^	− 1.55 (0.18)	− 1.92, − 1.2
	SWB → Workload → Emotion Recognition Difficulty → Perceived Stress	Ind3	0.01 (0.02)	− 0.04, 0.06
	Ind1—Ind2		1.07 (0.2)	0.68, 1.48
	Ind1—Ind3		− 0.49 (0.1)	− 0.68, − 0.31
	Ind2—Ind3		− 1.56 (0.19)	− 1.95, − 1.2
Work-related Burnout	SWB → Workload → Work Burnout	Ind1^	− 1.3 (0.28)	− 1.88, − 0.8
	SWB → Emotion Recognition Difficulty → Work Burnout	Ind2^	− 1.13 (0.5)	− 2.13, − 0.15
	SWB → Perceived Stress → Work Burnout	Ind3^	− 3.49 (0.49)	− 4.49, − 2.57
	SWB → Workload → Emotion Recognition Difficulty → Work Burnout	Ind4	< 0.01 (0.02)	− 0.03, 0.05
	SWB → Workload → Perceived Stress → Work Burnout	Ind5^	− 0.75 (0.16)	− 1.08, − 0.47
	SWB → Emotion Regulation Difficulty → Perceived Stress → Work Burnout	Ind6^	− 2.41 (0.34)	− 3.14, − 1.79
	SWB → Workload → Emotion Regulation Difficulty → Perceived Stress → Work Burnout	Ind7	0.01 (0.04)	− 0.06, 0.09
	Ind1—Ind2		− 0.18 (0.55)	− 1.26, 0.9
	Ind1—Ind3		2.19 (0.6)	1.01, 3.35
	Ind1—Ind5		− 0.56 (0.25)	− 1.09, − 0.08
	Ind1—Ind6		1.11 (0.47)	0.19, 2.01
	Ind2—Ind3		2.36 (0.75)	0.91, 3.79
	Ind2—Ind5		− 0.38 (0.53)	− 1.44, 0.66
	Ind2—Ind6		1.29 (0.61)	0.09, 2.51
	Ind3—Ind5		− 2.74 (0.51)	− 3.77, − 1.78
	Ind3—Ind6		− 1.08 (0.61)	− 2.26, 0.16
	Ind5—Ind6		1.66 (0.34)	1.04, 2.38
Student-related Burnout	SWB → Workload → Student Burnout	Ind1^	− 0.6 (0.27)	− 1.16, − 0.09
	SWB → Emotion Recognition Difficulty → Student Burnout	Ind2^	− 1.98 (0.76)	− 3.51, − 0.52
	SWB → Perceived Stress → Student Burnout	Ind3^	− 0.84 (0.41)	− 1.69, − 0.07
	SWB → Workload → Emotion Recognition Difficulty → Student Burnout	Ind4	0.01 (0.03)	− 0.06, 0.08
	SWB → Workload → Perceived Stress → Student Burnout	Ind5^	− 0.18 (0.09)	− 0.38, − 0.01
	SWB → Emotion Regulation Difficulty → Perceived Stress → Student Burnout	Ind6^	− 0.58 (0.28)	− 1.14, − 0.05
	SWB → Workload → Emotion Regulation Difficulty → Perceived Stress → Student Burnout	Ind7	0 (0.01)	− 0.02, 0.02
	Ind1—Ind2		1.38 (0.79)	− 0.16, 2.98
	Ind1—Ind3		0.24 (0.57)	− 0.86, 1.36
	Ind1—Ind5		− 0.42 (0.31)	− 1.03, 0.18
	Ind1—Ind6		− 0.02 (0.45)	− 0.9, 0.85
	Ind2—Ind3		− 1.14 (0.98)	− 3.06, 0.74
	Ind2—Ind5		− 1.8 (0.79)	− 3.39, − 0.28
	Ind2—Ind6		− 1.4 (0.89)	− 3.17, 0.33
	Ind3—Ind5		− 0.66 (0.34)	− 1.37, − 0.05
	Ind3—Ind6		− 0.26 (0.21)	− 0.75, 0.04
	Ind5—Ind6		0.4 (0.2)	0.03, 0.82

*p < .05, **p < .01, *** *p* < .001; ^ denotes significant indirect pathways; only pairwise comparisons between two significant indirect pathways are provided

#### Predicting work-related and student-related burnout

The conditional process model for predicting stress was then extended to assess the theorised downstream effects from stress to types of burnout in a further two analyses by making perceived stress a final predictor in the equation. The model depicted in Fig. 2B was found to predict work-related burnout well with 52% variance explained, *F*(10, 655) = 70.76, *p* < 0.001. A smaller portion of the variance in student-related burnout (Fig. 2C) was explained by these predictors at 17%, *F* (10, 655) = 13.19, *p* < 0.001. The regression statistics for the full models predicting work-related and student-related burnout are reported respectively in Table 4. Several differences were found between the two outcomes with regards to the importance of each variable.

When all steps had been added to the prediction of work-related burnout, four variables made significant independent contributions and, of these, perceived stress (*b* = 1.55; CI 1.32, 1.79) and workload (*b* = − 4.89; CI − 6.18, − 3.59) each explained large proportions of variance (13% and 5% respectively). Higher perceived stress and a more unmanageable workload were associated with higher work-related burnout. Years of teaching experience (*b* = − 0.12; CI − 0.22, − 0.03) and emotion regulation difficulties (*b* = 0.07; CI 0.01, 0.13) were also significant predictors of work-related burnout however, these associations were much lower (each < 1%).

Testing the relationship between subjective well-being and work-related burnout, the total effect *b* = − 10.33, *t*(666) = − 12.62, *p* < 0.001 of subjective well-being on work-related burnout was significant; however the direct effect *b* = 1.26, *t*(666) = − 1.49, *p* = 0.136 was not significant, suggesting that full mediation had occurred. The total indirect effect of subjective well-being on work-related burnout, representing the sum of the specific indirect effects, was significant, (*b* = − 9.07; CI 11.93, − 8.72).

When comparing indirect pathways from subjective well-being to work-related burnout, the consistently superior performing pathways (which were not significantly different from one another; see Table 5) were working through perceived stress or through emotion regulation difficulties and then through perceived stress.

While perceived stress was found to be the most important predictor of work-related burnout, its unique contribution to student-related burnout was significant, but smaller (< 1%). When all variables were considered together in predicting student-related burnout, we found a broader collection of contributors, though the contribution of these was small. The strongest predictors of student-related burnout were student-specific factors; being a primary or secondary teacher (*b* = − 8.12; CI 3.39, 12.85; *b* = 7.19; CI 2.71, 11.67 respectively). Being a teacher rather than holding a leadership position was associated with higher student-related burnout, with this accounting for approximately 1–2% of the variance. These educator type variables were not significant in the prediction of work-related burnout. The remaining significant predictors contributed approximately 1% of variance each and suggested that working in a rural or remote area (*b* = 8.01; CI 2.92, 13.10), perceiving workload as unmanageable (*b* = − 2.23; CI − 4.06, − 0.41), having greater emotion regulation difficulties (*b* = 0.13; CI 0.04, 0.21) and higher perceived stress (*b* = 0.37; CI 0.05, 0.70) were associated with greater student-related burnout.

Partial mediation was evident with the total *b* = − 6.61, *t* (666) = − 7.04, *p* < 0.001 and direct effects *b* = − 2.44, *t* (666) = − 2.04, *p* = 0.042 of subjective well-being on student-related burnout both being significant. The total indirect effect of subjective well-being on student-related burnout, representing the sum of the specific indirect effects, was significant (*b* = − 4.17; CI − 5.87, − 2.51).

Considering the indirect mediation paths between subjective well-being and student-related burnout, there were several equally performing mediation models. Indirect effects working through workload (Ind1), emotion regulation difficulties (Ind2), perceived stress (Ind3), and emotion regulation difficulties followed by perceived stress (Ind6) were not significantly different from one another, suggesting that the mediation model for student-related burnout is likely somewhat different from work-related burnout. Notably, the unique associations of emotion regulation difficulties and workload on both types of burnout existed in addition to their association via perceived stress. By contrast, subjective well-being had no independent association to burnout, with results indicating that perceived stress acts as a mediator between subjective well-being and both work-related and student-related burnout.

## Discussion

The present study aimed to add to the knowledge base about stress and burnout among Australian teachers. Data collected from a broad sample of current educators offered insights into the degree of stress and burnout across different demographic groups within the profession as well as the contributing role of intrapersonal (e.g., emotion regulation, subjective well-being) and environmental (e.g., excessive workload) factors. A concerningly large proportion (more than half) of teachers in this study reported finding their jobs either very or extremely stressful and were seriously considering leaving the profession. This high level of work-related distress in the sample concurs with other studies on teacher stress and attrition (e.g., Bottiani et al., 2019; Herman et al., 2018; OECD, 2020a), underscoring the urgency of understanding and addressing this important issue.

### Stress and burnout profiles across teacher characteristics

The variations in stress and burnout among groups of Australian teachers were explored. Results supported previous literature suggesting that early career teachers and those working within primary schools are at greatest risk of difficulties (e.g., Lau et al., 2005; Timms et al., 2006) but contradicted other evidence (Abel & Sewell, 1999) by suggesting that teachers in rural and remote areas experienced higher levels of stress and burnout than urban-based teachers.

High stress and burnout are common in the teaching profession generally, however early career teachers appear to be the most affected, as shown in this and many other studies (e.g., Goddard & Goddard, 2006; Plunkett & Dyson, 2011). This high rate of stress in early career teachers is driving their attrition from the profession at alarming rates. While there is some variation in reported attrition rates, in general the estimated rate of loss of early career teachers to the profession in many countries is approximately 40–50% within the first five years of teaching (Gallant & Riley, 2014). In Australia, estimates range more broadly between 8 and 50% (Queensland College of Teachers, 2016). Factors influencing this attrition in early career teachers are multiple and include heavy workload, the increasing complexity of teacher’s work, insufficient mentoring or support from leadership, and unstable patterns of employment (see Australian Institute of Teaching and School Leadership, AITSL, 2016).

Educator type or teaching level may be another factor contributing to stress, with results showing that primary school teachers were significantly more stressed than high school teachers in the present sample, however the generalisability of this outcome is unclear. For example, in an OECD report exploring whether teaching level correlated to experience of stress, no significant differences were found (OECD, 2020a). Within the Australian context, it may be warranted to consider the impact of national testing and data collection which is currently administered predominantly throughout the primary school years. A recent study of Australian teachers found that data collection and reporting were one of the main systemic sources of stress (Carroll, Flynn, et al., 2021). Another consideration is that the intensification of teaching may differentially affect primary and secondary school teachers (see Fitzgerald et al., 2019). Primary teachers are more generalist, have more face-to-face time with their students (McKenzie et al., 2014), and more contact with their students’ parents than secondary school teachers (Saltmarsh et al., 2015) all of which may potentially contribute to their levels of stress and burnout; more research is needed however.

In relation to burnout in particular, results of this study indicated that burnout rates were elevated for both primary and secondary classroom teachers relative to those in leadership roles. Furthermore, this was the case for both student-related burnout and work-related burnout. These findings are interesting to consider in light of a recent national survey which revealed that Australian school leaders (n = 2385) were physically and mentally distressed as a result of their job, with scores on domains such as stress, burnout, and sleep disturbance being nearly twice that of the general Australian population (Riley et al., 2020).

With regard to geographic location, the present study found that urban-based teachers experienced significantly lower levels of stress and burnout. Heightened stress in rural and remote areas may be the consequence of under-staffing and, by extension reduced access to resources and support (Plunkett & Dyson, 2011). Teachers outside of urban areas may experience personal and professional isolation, may be expected to fill more diverse roles due to under-staffing, and may have less access to professional development and other resources—all of which are likely to increase stress (Plunkett & Dyson, 2011).

The findings that both early career and rural/remote teachers appear to be at greater risk of stress and burnout, calls into question current teacher allocation practices in Australia. This practice often places early career teachers in regional, rural, and remote communities in the first years of their careers. Weldon’s (2018) review of Australian early career teacher attrition rates, also supports the notion that contextual factors such as location can play an important role in attrition and warrant better consideration when allocating early career teachers to schools. As highlighted in an independent review into regional, remote, and rural education, long-held beliefs about the country being the ideal place for beginning teachers may need to be challenged (Halsey, 2017).

### Predictors of stress and burnout in Australian teachers

This study also sought to explore the relative importance of emotion regulation, workload, and subjective well-being, on teacher stress and burnout. Together these predictor variables were found to account for a large proportion (approximately half) of the variance in both perceived stress and work-related burnout but explained substantially less variance in student-related burnout. The differences between the prediction of work-related and student-related burnout add support to the argument made by Kristensen and colleagues (2005) that these are distinct concepts and must be considered independently.

Emotion regulation and workload were notable in their contribution across all three outcomes, while the influence of subjective well-being, perceived stress and demographic variables fluctuated. The relative importance of each of these predictors are discussed in turn.

The ability to regulate emotions was found to have a major association with perceived stress, and also to have a unique association with burnout above and beyond its effect on perceived stress. Emotion regulation seemed to also partially account for the protective relationship of well-being on stress. This finding is in accordance with a wealth of research implicating emotion regulation as central to managing stress and general well-being (e.g., Jennings & Greenberg, 2009; Montgomery & Rupp, 2005). These results give strength to suggestions that stress-reduction interventions which focus on emotion regulation may be effective in reducing teacher stress. Indeed, several recent intervention studies focusing on emotion regulation have shown promise for improving stress and well-being for teachers (e.g., Carroll, Sanders O’Connor, et al., 2021; Flook et al., 2013).

Australian teachers’ perceptions of their workload were also found to be important to the development of stress and burnout. This outcome is not surprising given the frequency with which excessive workload is identified as a source of stress (e.g., Carroll, Flynn, et al., 2021; Kyriacou, 2001; Skaalvik & Skaalvik, 2015). In fact, the extent to which perceptions of an unmanageable workload increased stress and burnout appeared to be relatively unaffected by the association with emotion regulation. It is likely that holding such perceptions may have a direct relationship with stress by causing an imbalance between individual’s perceived external demands and their capacity, contributing to a sense of fatigue that is unaffected by that individual’s capacity for emotion regulation.

Interestingly, once the variables of workload and subjective well-being were added to the prediction of stress, several demographic factors were no longer relevant, suggesting that when teachers hold positive appraisals of their well-being and workload, this may protect them from stress regardless of school characteristics such as geographic location of the school, or level of students being taught. These results echo findings from the OECD (2020a) report on teachers and school leaders. Although the report noted that workload was one of the most prominent sources of stress for teachers, more detailed analysis revealed that variance in teachers’ reported workload stress was overwhelmingly accounted for by differences among teachers within schools (97%) rather than differences between schools such as school composition or location (3%). This suggests that intrinsic traits of individual teachers such as their resilience, emotion regulation capabilities and sense of subjective well-being, may play a significant role in the experience of workplace stress. Caution needs to be heeded however in over-simplifying interpretation of these findings. Given the frequency with which workload is identified as a major stressor, paired with the low variance between-schools, this may suggest that workload issues are a systemic problem and need to be addressed at a policy level within education departments and jurisdictions. Overall, while these findings require replication, they provide preliminary support for suggestions that interventions aimed at reducing teacher stress may be effective across all types of teacher groups if they include strategies that address workload.

Teachers’ subjective well-being was found to be among the most important predictors of perceived stress, whereby higher levels of subjective well-being were associated with lower stress, independent of workload and emotion regulation skills. Such a finding corresponds with a large body of evidence indicating that subjective well-being is central to psychological and physical well-being (Diener & Chan, 2011), and provides support for the suggestion that interventions which directly target well-being may be central to attempts to reduce teacher stress (Jennings & Greenberg, 2009). However, beyond its association with stress, subjective well-being had a unique influence on student-related burnout but not on work-related burnout. Results suggest that perceived stress may act as a partial mediator between subjective well-being and student-related burnout, and a full mediator for work-related burnout. This pattern aligns with the “burnout cascade” described by Jennings and Greenberg (2009) whereby teachers’ well-being indirectly influences their emotional exhaustion via the classroom climate; poor well-being leads to a poor classroom climate, which in turn increases exhaustion and burnout.

The obtained results suggest that a more precise prediction of student-related burnout requires variables beyond those measured in this study. Among the included variables, role-specific factors such as being a classroom teacher relative to being in an administrative role, and perceptions of workload were the strongest predictors of student-related burnout. Interestingly, while perceived stress was the strongest predictor of work-related burnout, it had a very small influence on student-related burnout. Such a result defies the theorised pathway from stress to burnout, suggesting that student-related burnout may be a uniquely different construct to other forms of burnout. Factors such as student behaviour, classroom climate, and teacher self-efficacy are likely to be stronger predictors of burnout specifically related to students.

### Limitations and conclusions

The present study is not without its limitations. Recognising that it relies on self-report methodology which can be susceptible to subjective bias, future research would benefit from collecting more objective measures of stress (e.g., cortisol) and workload (e.g., task logs). It is also acknowledged that the reliability for the Workload scale was slightly lower than other scales. As such, findings need to be interpreted with some caution and future research is needed to replicate the findings with a larger sample size. Moreover, this study used cross-sectional data from one country and it would therefore be beneficial to replicate the study in other contexts. Future research should also consider assessing other intrapersonal factors such as personality traits, which have been implicated as important in assessing vulnerability for stress and work-related burnout (see Kokkinos, 2007 for review) and should include a broader set of factors in the prediction of student-related burnout. While not the focus of the present research, a supportive leadership team has been found to be a key protective factor for teachers as are teaching communities of support (Granziera et al., 2021). Such factors would be of value to include in future research. It should also be noted that this study was conducted prior to the COVID-19 pandemic that added significant extra pressure to an already stretched teaching workforce. Teachers have faced heightened stress in remaining in the workplace and/or pivoting to online learning during lockdowns, providing an in-place education to vulnerable students, and navigating the lived experience of students. These factors which have likely exacerbated teacher stress and burnout, although this would need to be further assessed.

The present study has contributed to the field by providing new insights into the stress and burnout experienced by various demographic groups of Australian teachers and highlighting that emotion regulation, workload and subjective well-being play a role in the degree of stress and burnout they experience. Teachers play a crucial role in the development of our future generations and their well-being is central to their ability to effectively fulfil this role, yet in the present study over half of the sample reported being very or extremely stressed, with early career teachers, primary teachers, and teachers working in rural and remote areas reporting the highest stress and burnout levels. The results of this study underscore the stress and burnout burdening many Australian teachers and emphasise the urgent need for multifaceted interventions to reduce this burden in a post-COVID world. Interventions that aim to address the manageability of teacher’s workload, improve their capacity for effective emotion regulation, and improve their sense of subjective well-being are likely to provide substantial benefits to teachers and support their healthy and successful functioning at work.
